# A variant W chromosome in *Centromochlus heckelii* (Siluriformes, Auchenipteridae) and the role of repeated DNA in its heteromorphism

**DOI:** 10.1590/1678-4685-GMB-2024-0071

**Published:** 2025-01-27

**Authors:** Chrystian Aparecido Grillo Haerter, Patrik Ferreira Viana, Fábio Hiroshi Takagui, Sandro Tonello, Vladimir Pavan Margarido, Daniel Rodrigues Blanco, Josiane Baccarin Traldi, Roberto Laridondo Lui, Eliana Feldberg

**Affiliations:** 1 Instituto Nacional de Pesquisas da Amazônia, Programa de Pós-Graduação em Genética, Conservação e Biologia Evolutiva (PPG GCBEv), Manaus, AM, Brazil.; 2 Universidade Estadual do Oeste do Paraná, Centro de Ciências Biológicas e da Saúde (CCBS), Cascavel, PR, Brazil.; 3 Universidade Tecnológica Federal do Paraná, Coordenação de Ciências Biológicas (COBIO), Campus Santa Helena, Paraná, PR, Brazil.

**Keywords:** Auchenipteridae cytogenetics, heteromorphic sex chromosomes, interstitial telomeric sequence, repetitive DNA mapping.

## Abstract

*Centromochlus heckelii* has the lowest diploid chromosome number (2n = 46) and the only described heteromorphic sex chromosome system in Auchenipteridae. This study presents a population of *C. heckelii* from the Central Amazon basin with subtle variations in the karyotype composition and a variant W chromosome with distinct morphology and increased C-positive heterochromatin content. In this population, the W chromosome is subtelocentric, whereas the only previous study on *C*. *heckelii* reported a metacentric W chromosome. Constitutive heterochromatin (CH) and accumulation of microsatellite motifs have significantly contributed to this W chromosome enlargement. Notably, this population exhibits numerous interstitial telomeric sites (ITSs). Some of these ITSs might represent genuine chromosomal fusion points due to the reduced 2n; however, additional mechanisms, such as chromosomal inversions, translocations, transpositions, or association with satellite DNA, are likely responsible for this unusual pattern. The 18S rDNA sites were found in both the Z and W chromosomes of all individuals. However, two individuals exhibited an additional 18S rDNA site in a single homologous of the chromosome pair 20, characterizing an intrapopulation polymorphism. The 5S rDNA sites were found in two chromosome pairs, distinguishing this population from other Centromochlinae species and further supporting it as one of the most efficient cytotaxonomic markers within the subfamily.

## Introduction

The Auchenipteridae family, commonly referred to as the driftwood catfishes, comprises 25 genera and 128 species (Calegari et al., 2019; Sarmento-Soares and Martins-Pinheiro, 2021; Fricke *et al.*, 2024). This monophyletic group is distributed throughout the Neotropical region, ranging from Argentina to Panama, and inhabits diverse freshwater habitats, such as streams, rivers, and lakes, commonly found in submerged logs (Calegari *et al.*, 2019). Auchenipteridae is currently organized into two well-supported monophyletic clades: the subfamilies Auchenipterinae and Centromochlinae. However, the relationships among genera and species within both subfamilies have long been a source of inconsistencies (see Calegari *et al.*, 2019; Sarmento-Soares and Martins-Pinheiro, 2021). According to the most recent phylogenetic analysis (Calegari *et al.*, 2019), Auchenipterinae comprises 78 species and 18 genera. Conversely, two recent phylogenetic studies proposed different classifications for Centromochlinae (i.e., Calegari *et al.*, 2019; Sarmento-Soares and Martins-Pinheiro, 2021). As a result, Centromochlinae currently comprises seven genera with 50 valid species (Fricke *et al.,* 2024), but the relationships are still a point of debate.

Auchenipteridae species are characterized by a recurrent diploid chromosome number (2n) of 58 chromosomes, with minimal variations in the karyotype composition (summarized in Kowalski et al., 2024). A few exceptions are present among the cytogenetically analyzed species: *Ageneiosus inermis* (Linnaeus, 1766) (cited as *Ageneiosus brevifilis*) and *Tympanopleura atronasus* (Eigenmann and Eigenmann 1888) (Fenocchio and Bertollo, 1992; Lui et al., 2013a) with 2n = 56, and *Tetranematichthys wallacei* Vari and Ferraris 2006 with 2n = 52 (Casarotto et al., 2024). The most recent noteworthy example is *Centromochlus heckelii* De Filippi 1853, which possesses the lowest diploid chromosome number (2n = 46), the only multiple nucleolar organizer regions (NORs), and the only described heteromorphic sex chromosome system in Auchenipteridae so far (Kowalski *et al.*, 2020). The heteromorphic chromosome was found in females, characterizing a ZZ/ZW sex chromosome system in which the W chromosome is metacentric, almost entirely heterochromatic, and the largest chromosome of the karyotype (Kowalski *et al.*, 2020). 

Heteromorphic sex chromosomes are highly diverse among Neotropical freshwater fish (reviewed in Cioffi et al., 2017; Sember et al., 2021). The canonical model posits that suppression of meiotic recombination between X and Y or Z and W chromosomes is the first step and a precondition for genetic and morphological differentiation (Charlesworth et al., 2005; Graves, 2006; Bachtrog, 2013; Schartl et al., 2016; Wright et al., 2016; Furman et al., 2020; Charlesworth, 2021). Because of recombination suppression, the sex-limited chromosome can suffer genetic degeneration, heterochromatinization, and accumulation of repetitive DNA, which, over time, can result in significant differences in size, gene content, and severely degenerated W or Y chromosomes (Charlesworth *et al.*, 2005; Graves, 2006; Bachtrog, 2013; Schartl *et al.*, 2016; Wright *et al.*, 2016; Furman *et al.*, 2020; Charlesworth, 2021). Identifying these evolutionary mechanisms is an essential step for comprehending the differentiation process and evolution of heteromorphic sex chromosomes. However, only one population of *C. heckelii* was studied through cytogenetic methods, including only Giemsa staining, C-banding, and detection of NORs using silver nitrate impregnation (Ag-NORs). Consequently, information regarding the W chromosome differentiation, 2n reduction, and rDNA organization remains limited in this species.

This study investigates karyotypic diversification and sex chromosome differentiation in *C. heckelii* using a combination of conventional and molecular cytogenetic markers. We aimed to disentangle the mechanisms of chromosome number reduction and to identify the repeated sequences, if any, involved in these processes and in the sex chromosome differentiation of this species. Telomeric probes, which can reveal putative points of chromosomal fusion (reviewed in Vicari et al., 2022), were used to investigate the 2n reduction. Ribosomal DNA probes (18S and 5S) were used to investigate sex chromosome differentiation because the only previous study reported these cistrons on the sex chromosomes (Kowalski et al., 2020), and a syntenic rDNA pattern was identified in *C. schultzi*, a closely related species (Kowalski *et al.*, 2024). Furthermore, the 5S rDNA is a valuable cytotaxonomic marker in Auchenipteridae (Kowalski *et al.*, 2024) and might contribute to the taxonomic issues within Centromochlinae. Finally, SSRs were specifically used to investigate the W chromosome differentiation. This type of repetitive sequence tends to accumulate on the sex-limited chromosome when recombination is suppressed (Bachtrog, 2013; Schartl et al., 2016; Wright et al., 2016; Furman et al., 2020) and has been widely used to study sex chromosome systems in Neotropical fish [overview in Haerter et al. (2023)]. Thus, we expect that similar sequences may be present in the W chromosome of *C. heckeelii*.

## Material and Methods

### Sampling and mitotic chromosome obtaining

We analyzed 22 individuals of *C. heckelii* (10 males and 12 females) from the Amazonas River, near Furo do Paracuúba-AM (3°12’37.1”S; 59°59’10.6”W). The individuals were euthanized by clove oil overdose (Griffiths, 2000), according to the Ethics Committee on the Use of Animals (CEUA) of the Instituto Nacional de Pesquisas da Amazônia (INPA) (SEI 01280.001883/2022-52). Mitotic chromosomes were obtained from anterior kidney cells, according to Gold et al. (1990). The animals were collected under the permits granted by the Instituto Chico Mendes de Conservação da Biodiversidade (ICMBio) (permit numbers 49379, 28095, and 84946) and deposited at the fish collection of the INPA (voucher ID: INPA-ICT 059876).

### Conventional cytogenetics 

The chromosomes were stained with 5% Giemsa solution diluted in phosphate buffer (pH = 6.8) and classified based on their arm ratio, according to Levan et al. (1964). C-positive heterochromatin detection was performed according to Sumner (1972), and the chromosomes were stained with propidium iodide (Lui et al., 2012). In addition to the morphology, the sex chromosomes of this species were characterized by the presence of NORs (Kowalski et al., 2020). Therefore, we used silver nitrate impregnation (Ag-NORs; Howell and Black, 1980) to identify the sex chromosomes after FISH under a light microscope.

### Repetitive DNA probes 

The 18S rDNA probes were obtained from a mini-prep of *Prochilodus argenteus* Spix and Agassiz 1829 (Hatanaka and Galetti, 2004). The 5S rDNA probes were obtained from a mini-prep of *Megaleporinus elongatus* Valenciennes 1850 (Martins and Galetti, 1999). The 18S probes were labeled by nick-translation with biotin-16-dUTP (green), according to the manufacturer’s protocol (Bio-Nick-Translation Mix, Roche Diagnostics, Mannheim, Germany). The 5S rDNA probes were labeled with digoxigenin-11-dUTP (red) using the same method, according to the manufacturer’s protocol (Dig-Nick-Translation Mix, Roche Diagnostics). 

The telomeric probes were isolated and labeled by polymerase chain reaction (PCR) using a pair of self-complementary telomeric primers described by Ijdo et al. (1991). The PCR reaction was composed of 1x PCR buffer, 1.5 mM of MgCl_2_, 0.2 mM of dNTPs mix, 1 µM of each primer, 0.5 U of *Taq* DNA polymerase (Roche Diagnostics), 0.025 mM of tetramethyl-rhodamine-5-dUTP (red; Roche Diagnostics), and PCR-grade water to fill a final volume of 12.5 µl. The PCR conditions were as follows: 95 °C (1 min); 10 cycles of 95 °C (1 min), 55 °C (30 s), and 72 °C (1 min); 30 cycles of 95 °C (1 min), 60 °C (30 s), and 72 °C (30 s); and a final extension at 72 °C (1 min). 

The SSRs (AG)_n_, (AC)_n_, (AGC)_n_, (AAT)_n_, (GGAT)_n_, and (GATA)_n_ were directly labeled with Cy-3 during the synthesis. They were chosen based on their recurrence in previous studies of heteromorphic sex chromosomes in Neotropical fish [summarized in Haerter et al. (2023)].

### Fluorescence *in situ* hybridization (FISH)

The FISH experiments were carried out according to Yano et al. (2017a) with minor modifications. Briefly, the slides pre-treatment included: (a) incubation in RNAse for 1 h at 37 ºC in a moist chamber; (b) two washes in 2x saline-sodium citrate buffer (SSC) for 5 min each, pH = 7.0; (c) dehydration by ethanol series of 70 and 100% at room temperature, 5 min each; (d) denaturation of the chromosome DNA on the slides in 70% deionized formamide/2x SCC at 70 ºC; (e) dehydration by ethanol series of 70 and 100% at -20 ºC. The hybridization mixture was composed of 150-200 ng of each probe, 50% formamide, 10% dextran sulfate, 2x SSC, pH = 7.0-7.2. The denaturation of the hybridization mixture was performed in a dry block at 99 ºC for 10 min. After denaturation, the hybridization mixture was submitted to thermal shock on ice, preserving the probes as single-stranded DNA. The hybridization mixture was then applied to each slide and incubated with a coverslip overnight at 37 ºC in a moist chamber. 

Post-hybridization washes were carried out as follows: 10 min in 15% deionized formamide/2x SSC at 42 ºC and thrice in 0.5% tween/4x SSC at room temperature for 5 min each. The 5S rDNA probes were detected using anti-digoxigenin-rhodamine (Roche Diagnostics). The 18S rDNA probes were detected using avidin-FITC. The signal was amplified by incubation with biotinylated anti-avidin (Roche Diagnostics) and a second round of incubation with avidin-FITC (Roche Diagnostics). After brief air-drying, the slides were counterstained with DAPI (4’,6-Diamidino-2-Phenylindole, 1.2 µg/mL) and mounted in Vectashield anti-fade medium (Vector Laboratories, California, United States).

### Karyotypic and microscopic analyses

The images were captured by the DP Controller 3.2.1.276 software using a Olympus DP71 digital camera connected to a BX61 epifluorescence microscope (Olympus America Inc., Center Valley, PA, United States of America). The images were assembled using the GIMP image editor. Interpretations were based on 10-20 metaphases per individual analyzed and techniques performed. All individuals were analyzed using Giemsa staining, C-banding, telomeric probes, and 18S rDNA. The 5S rDNA and telomeric probes were applied to ten of our best metaphase-quality individuals (five males and five females). SSRs were hybridized in six of the best metaphase-quality individuals (three males and three females). The number of individuals per analysis was determined based on the consistency of the patterns observed during the analyses. We expanded the analyse to more individuals for chromosomal markers that exhibited variable patterns. Those included the 18S rDNA, the CH content, and the distinct morphology of the W chromosome.

### Ethical approval

The animals were collected under permits granted by the Instituto Chico Mendes de Conservação da Biodiversidade (ICMBio) (permit numbers 49379, 28095, and 84946) and euthanized following the guidelines of the Ethics Committee on the Use of Animals (CEUA) of the Instituto Nacional de Pesquisas da Amazônia (INPA) (SEI 01280.001883/2022-52). 

## Results

**Figure 1-:**
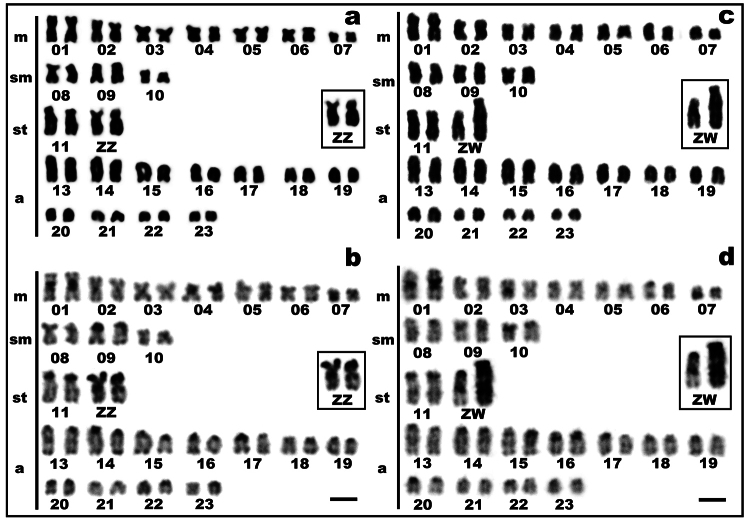
Karyotype of male **(a, b)** and female **(c, d)** of *C. heckelii* stained with Giemsa **(a, c)** and sequentially subjected to C-banding analysis **(b, d)**. The sex chromosomes (Z and W) are shown in the boxes. m = metacentric; sm = submetacentric; st = subtelocentric; a = acrocentric. Scale bar = 5 µm.

**Figure 2-:**
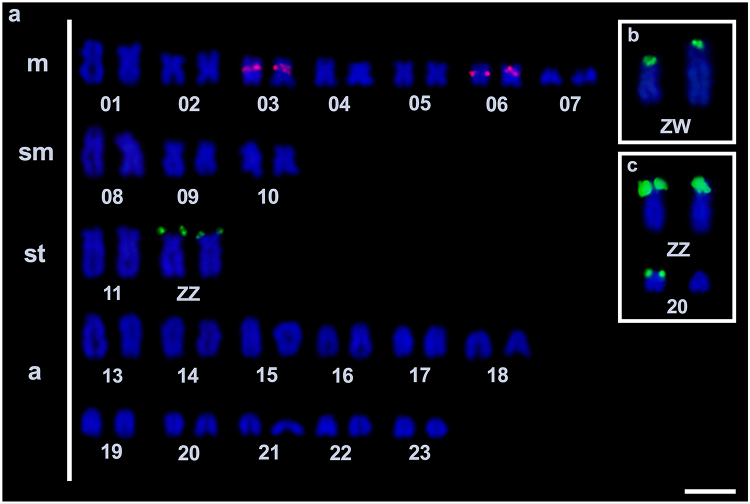
Karyotype of *C. heckelii* hybridized with 18S rDNA probes (green) and 5S rDNA probes (red) and counterstained with DAPI (blue). The heteromorphic sex chromosome pair of the female (ZW) is shown in the box (b). The polymorphic pattern, including three 18S rDNA sites (ZZ + one of the homologous chromosomes of pair 20a), is shown in the box (c). m = metacentric; sm = submetacentric; st = subtelocentric; a = acrocentric. Scale bar = 5 µm.

**Figure 3-:**
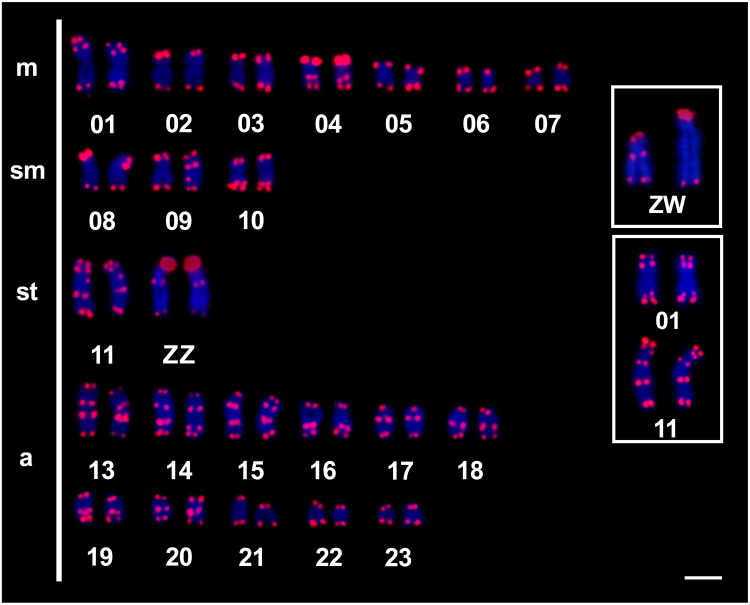
Karyotype of *C. heckelii* hybridized with telomeric probes (red) and counterstained with DAPI (blue). The heteromorphic sex chromosome pair of the female (ZW) is shown in the box. Improved signal quality of the ITSs found in the chromosome pairs 01 and 11 is also shown in the box. m = metacentric; sm = submetacentric; st = subtelocentric; a = acrocentric. Scale bar = 5 µm.

This *C. heckelii* population from Furo do Paracuúba had a diploid chromosome number of 46 for both males and females, with a karyotype composed of 14 metacentric (m), 6 submetacentric (sm), 4 subtelocentric (st), and 22 acrocentric (a) chromosomes (Figure 1). Females had a heteromorphic chromosome pair, characterizing a ZZ/ZW heteromorphic sex chromosome system. The Z chromosome is subtelocentric and presents terminal and interstitial CH. The W is subtelocentric, the largest chromosome in the karyotype, and has a significant accumulation of CH (Figure 1 c, d). The 18S rDNA sites were detected at the terminal position of the short arm of chromosome pair 12(st) (ZZ in males and ZW in females; Figure 2 a, b). Additionally, the 18S rDNA was detected in the ZZ chromosomes and in one homologous chromosome of pair 20(a) in two males (Figure 2 c). The 5S rDNA sites were detected at the proximal position of the short arm of chromosome pairs 3(m) and 6(m) (Figure 2 a). Telomeric sequences were detected at the telomeric position of all chromosomes; however, interstitial telomeric sequences (ITSs) were also found in 14 chromosome pairs (Figure 3). Four chromosome pairs had two ITSs (1, 13, 14, and 15), and one chromosome pair had three ITSs (pair 11). Notably, the Z chromosome had one ITS, and the W chromosome had none. 

The C-positive heterochromatin was found on centromeric and terminal positions of most chromosomes (Figure 1 b , d). Most biarmed chromosomes showed a preferential accumulation of CH at the centromeric position, while the acrocentric chromosomes exhibited CH preferentially accumulated at the terminal position. Interstitial CH (proximal and distal) was also observed in some chromosomes (e.g., chromosome pairs 11, 13, 14, and 15). The Z chromosome had a prominent CH block at the proximal position of the long arm and at the 18S rDNA position (Figure 1 b ). The W chromosome was almost entirely heterochromatic (Figure 1 d ).

**Figure 4 -:**
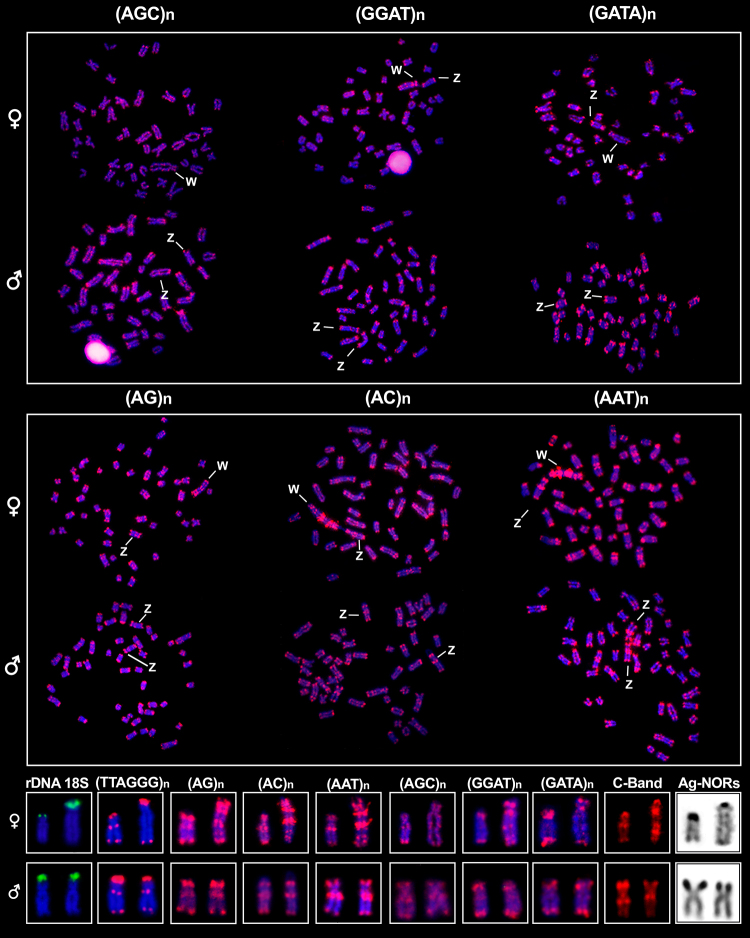
Metaphase plates of *C. heckelii* hybridized with the microsatellite probes (AG)_n_, (AC)_n_, (AAT)_n_, (AGC)_n_, (GGAT)_n_, and (GATA)_n_ (red) and counterstained with DAPI (blue). An overview of all repetitive markers used in this study that hybridized on the sex chromosomes is presented in the boxes below the metaphase plates. An inset of the Ag-NORs detected in the sex chromosomes is also presented. Scale bar = 5 µm.

The mapping of all six SSRs-(AG)_n_, (AC)_n_, (AGC)_n_, (AAT)_n_, (GGAT)_n_, and (GATA)_n_-revealed hybridization signals throughout the chromosomes in a consistent pattern (Figure 4), primarily located at the terminal region of both chromosome arms and at the interstitial positions of nearly all chromosomes. This same pattern was also observed in the Z chromosome. However, the Z chromosome also displayed an additional large block of SSRs at the proximal position of the long arm, coinciding with the C-positive heterochromatin block. In contrast, the W chromosome had a notable accumulation of all mapped SSRs (Figure 4).

## Discussion

This population of *C. heckelii* from Furo do Paracuúba has 46 chromosomes, an accumulation of SSRs on the W chromosome, and several ITSs. Additionally, it has a W chromosome with different morphology and C-banding pattern compared to the previously and only analyzed population of *C. heckelii*, sampled in the Amazon River (Kowalski et al., 2020). 

Due to the reduced 2n, some ITSs may genuinely represent remnants of chromosomal fusions in *C. heckelii*. However, this species has an elevated number of ITSs, suggesting that additional mechanisms contributed to this unusual pattern, including chromosomal inversions, translocations (Bolzán, 2017), ectopic transpositions (Scacchetti et al., 2015; Oliveira et al., 2021), or association with satellite DNA (Bolzán, 2017). Among these mechanisms, chromosomal inversions seem to represent a major source of ITSs in *C. heckelii* since there is a significant reduction in biarmed chromosomes compared to *C. schultzi* (Kowalski et al., 2024). Chromosomal inversions can move telomeric segments to the interstitial position of the chromosomes, where they can be amplified multiple times, resulting in ITSs detectable through cytogenetic methods without changing the 2n (reviewed in Bolzán, 2017). This mechanism is also the most parsimonious explanation for the heterochromatic ITSs (Het-ITSs) in *C. schultzi* since this species had no 2n reduction (Kowalski *et al.*, 2024).

The variation in the W chromosome between the Amazon River population (Kowalski et al., 2020) and our sample suggests that *C. heckelii* has an intraspecific polymorphism of the sex-limited chromosome. Although most studies compare the homologous sex chromosomes (male *vs.* female) or sex chromosome systems across different species (summarized in Sember et al., 2021), variations can also occur between populations of the same species. Intraspecific variation is reported in several vertebrate taxa, including well-studied fish models such as *Nothobranchius furzeri* Jubb 1971 and *Poecilia reticulata* Peters 1859 (reviewed in Furman et al., 2020). It can range from small differences in non-recombining regions to substantial heterochromatin and chromosomal structural variations (Furman *et al.*, 2020). Particularly for young sex chromosomes of fish (Schartl et al., 2016), the intraspecific diversity can be high since it takes time for variants to become fixed, leading to periods of polymorphism (Furman *et al.*, 2020). In *C. heckelii*, the widespread distribution throughout the Amazon and Orinoco River basins may be delaying the fixation of a specific W chromosome form or allowing the emergence of different W chromosome forms since this is not a recently diverged species [estimated divergence time: 2.40-3.43 MYA; based on Timetree.org using the dataset of Cassemiro et al. (2023)].

The heterochromatinization and heterochromatin amplification observed on the W chromosome of *C. heckelii* is a recurring process in the differentiation of sex chromosomes in Neotropical fish species, especially in ZZ/ZW systems (e.g., Vissotto et al., 1997; Cioffi et al., 2017; Takagui et al., 2017; Yano et al., 2017b; Kowalski et al., 2020). Although the heterochromatin content in the genome is regulated by several biological and biochemical processes (reviewed in Allshire and Madhani, 2018), genetic alterations cannot be purged in the absence of recombination in the sex-specific region (Schartl et al., 2016). Consequently, the sex-limited chromosome can become highly heterochromatic and experience high levels of gene loss even though the X or Z chromosome remains functional (Rice, 1987; Bachtrog, 2013; Wright et al., 2016). Interestingly, the population of the Amazon River (Kowalski *et al.*, 2020) and Furo do Paracúúba also exhibit slight differences in CH content on the W chromosome. This type of differential amplification or contraction of the CH-linked repeats is a recurrent feature among populations (e.g., Blanco et al., 2010; Hashimoto and Porto-Foresti, 2010; Baümgartner et al., 2014) and can lead to notable polymorphisms in sex chromosomes (e.g., Felip et al., 2004; Cioffi *et al.*, 2012b; Nanda et al., 2014; Yano *et al.*, 2021). These differences in the CH content of the W chromosome of *C. heckelii* may suggest a polymorphic state; however, we should not rule out the possibility of different levels of chromosomal contraction affecting the C-banding analyses of both studies.

The W chromosome of this *C. heckelii* population exhibits an accumulation of SSRs, a recurring feature not only observed in fish but also in various other organisms (see Kejnovsky et al., 2009; Ezaz and Deakin, 2014; Cioffi et al., 2012 a ; 2017). This process is primarily attributed to lower purifying selection on the sex-linked region, which allows repeated DNA, mainly SSRs and transposons, to rapidly accumulate (see Reichwald et al., 2015; Schartl et al., 2016; Furman et al., 2020). In *C. heckelii*, the Z chromosome also has a significant accumulation of SSRs. This arrangement might have facilitated the differentiation of the ancestral homomorphic sex chromosomes through differential expansion of the pre-existing SSR blocks on the W chromosome (for mechanisms, see Ellegren, 2004; Oliveira et al., 2006; Kalia et al., 2011). A similar accumulation of the microsatellite (GATA)_n_ in other Auchenipteridae species demonstrates that this scattered pattern is an ancestral condition of the family and not an exclusive pattern of *C. heckelii* (Lui et al., 2021; Felicetti et al., 2021; Haerter et al., 2023). Furthermore, there is a considerable overlap between the mapped SSRs and the CH regions of *C. heckelii*. Heterochromatin regions often harbor repetitive elements, including satellite repeats and transposable elements (Allshire and Madhani, 2018). Thus, this overlap between the mapped SSRs and CH areas suggests they correspond to CH-linked repeats. However, their participation in the W chromosome polymorphism remains unclear because the Amazon River population was only analyzed using conventional cytogenetics (Kowalski et al., 2024). 

*Centromochlus heckelii* was previously described with multiple NORs, including sites on chromosome pairs 21(a) and ZZ/ZW (Kowalski et al., 2020). However, no differences in 18S rDNA distribution were found between the sex chromosomes, suggesting that this sequence may not be involved in their differentiation. Most individuals display the typical Auchenipteridae 18S rDNA pattern: a single site at the terminal position of a subtelocentric chromosome (reviewed in Kowalski *et al.*, 2024). Despite this, our sample has a numeric variation of the 18S rDNA sites, without correlation with the sex, characterized by individuals with three sites while others exhibit only two. This type of numerical 18S rDNA polymorphism is well-documented in various fish groups, such as *Symphysodon* (Gross et al., 2010), *Hypostomus* (Traldi et al., 2013; Oliveira et al., 2019), *Hoplias* (Oliveira *et al.*, 2015), *Oligosarcus* (Usso et al., 2018), and *Astyanax* (Tonello et al., 2022). Numerous mechanisms can cause this variation, such as the entire deletion of the rDNA cluster from one of the homologous chromosomes or its transposition to a new location (e.g., Cabrero and Camacho, 2008; Raskina et al., 2004, 2008), loss of sequence in one of the homologous chromosomes due to unequal recombination during crossing-over, or chromosomal translocations (e.g., Cabrero and Camacho, 2008; Nguyen et al., 2010; Cabral-de-Mello et al., 2011; Takagui et al., 2023). Alternatively, silver nitrate can also stain proteins unrelated to NORs, and this “pseudo NOR site” is therefore not detected with 18S rDNA probes (e.g., Nirchio et al., 2007).

On the other hand, the 5S rDNA remains one of the most variable and efficient cytotaxonomic markers within Auchenipteridae. The number of sites in *C. heckelii* can be used to differentiate it from other Centromochlinae species: *G. ribeiroi* (one site; Lui et al., 2015), *T. neivai* (three sites; Lui *et al.*, 2013b), *T. jaracatia* (four sites; Lui *et al.*, 2013b), and *C. schultzi* (four sites; Kowalski et al., 2024). Notably, the synteny of 18S and 5S rDNA is a recurrent feature in Doradidae (Takagui et al., 2021), the sister group of Auchenipteridae (Sabaj and Arce, 2021). However, *C. schultzi* (Kowalski *et al.*, 2024) is the only Auchenipteridae species reported with this syntenic pattern. Given the lower phylogenetic distance, it was expected that *C. heckelii* could also have this synteny. However, the 5S and 18S rDNA were found in distinct chromosome pairs, suggesting that the 5S rDNA has a highly variable pathway in Auchenipteridae. Interestingly, *C. schultzi* is a former species of the extinct genus *Bauroglanis* (see Calegari et al., 2019; Sarmento-Soarez and Martins-Pinheiro, 2021), and this syntenic pattern was suggested as a possible cytotaxonomic marker, potentially an apomorphy of the species or a synapomorphy of some species (Kowalski *et al.*, 2024). The absence of this characteristic in *C. heckelii*, the type species of *Centromochlus*, is congruent with this hypothesis, but the analysis of additional species is still needed to infer its efficiency. Usually, these types of cytogenetic variations can constitute excellent cytogenetic markers, providing important insights into differentiation trajectories of fish karyotypes (Gornung, 2013; Ocalewicz, 2013), especially in groups with taxonomies not yet fully consolidated, such as Auchenipteridae.

*Centromochlus heckelii* has the lowest 2n and the only described heteromorphic sex chromosome system within Auchenipteridae thus far. Some characteristics stand out in this species, including: a) the presence of intraspecific variation in the W chromosome; b) the reduced 2n and the presence of ITSs suggest that chromosomal fusions have occurred. However, this species has a large number of ITSs, which hinders precise identification of the fused chromosome pairs; c) while some of the ITSs may represent genuine fusion points, most of them have probably originated through other mechanisms, such as chromosomal inversions, translocations, transpositions, or association with satellite DNA; d) repetitive DNA and heterochromatin accumulation have a prominent role in the differentiation of the sex chromosomes of this species.
